# Highly Effective Anti-Organic Fouling Performance of a Modified PVDF Membrane Using a Triple-Component Copolymer of P(St_x_-*co*-MAA_y_)-*g*-fPEG_z_ as the Additive

**DOI:** 10.3390/membranes11120951

**Published:** 2021-11-30

**Authors:** Xiaoji Zhou, Yizhuo Sun, Shusu Shen, Yan Li, Renbi Bai

**Affiliations:** 1 Center for Separation and Purification Materials & Technologies, Suzhou University of Science and Technology, Suzhou 215009, China; zhou-xiaoji@163.com (X.Z.); sunyizhuo2019@163.com (Y.S.); shusushen@mail.usts.edu.cn (S.S.); liyan370403@163.com (Y.L.); 2 School of Environmental Science and Engineering, Suzhou University of Science and Technology, Suzhou 215009, China; 3 Jiangsu Collaborative Innovation Center for Technology and Material of Water Treatment, Suzhou 215009, China

**Keywords:** a triple-component copolymer additive, modified PVDF membrane, hydrophilic and oleophobic surface property, low protein and organic fouling

## Abstract

In this study, a triple-component copolymer of P(St_x_-*co*-MAA_y_)-*g*-fPEG_z_ containing hydrophobic (styrene, St), hydrophilic (methacrylic acid, MAA), and oleophobic (perfluoroalkyl polyethylene glycol, fPEG) segments was synthesized and used as an additive polymer to prepare modified PVDF membrane for enhanced anti-fouling performance. Two compositions of St:MAA at 4:1 and 1:1 for the additive and two blending ratios of the additive:PVDF at 1:9 and 3:7 for the modified membranes were specifically examined. The results showed that the presence of the copolymer additive greatly affected the morphology and performance of the modified PVDF membranes. Especially, in a lower ratio of St to MAA (e.g., St:MAA at 1:1 versus 4:1), the additive polymer and therefore the modified PVDF membrane exhibited both better hydrophilic as well as oleophobic surface property. The prepared membrane can achieve a water contact angle at as low as 48.80° and display an underwater oil contact angle at as high as 160°. Adsorption experiments showed that BSA adsorption (in the concentration range of 0.8 to 2 g/L) on the modified PVDF membrane can be reduced by as much as 93%. From the filtration of BSA solution, HA solution, and oil/water emulsion, it was confirmed that the obtained membrane showed excellent resistance to these organic foulants that are often considered challenging in membrane water treatment. The performance displayed slow flux decay during filtration and high flux recovery after simple water cleaning. The developed membrane can therefore have a good potential to be used in such applications as water and wastewater treatment where protein and other organic pollutants (including oils) may cause severe fouling problems to conventional polymeric membranes.

## 1. Introduction

Polyvinylidene fluoride (PVDF), attributed with excellent chemical stability and engineering processability, has been used as one of the preferred materials to prepare polymeric membranes for water and wastewater treatment [1]. However, the strong intrinsic hydrophobicity of the PVDF material often causes membranes with severe organic fouling, a phenomenon of rapid and often irreversible loss in the permeate flux [2,3,4], which has greatly limited their prospect for more widespread applications [5,6,7]. Organic foulants can tightly attach to the external or internal surfaces of the membranes (due to some extent of physicochemical interactions) and not be easily or effectively removed by simple physical cleaning (such as water washing), leading to the use of more expensive and even tedious chemical cleaning strategy [8,9]. Thus, the cost of membrane applications in water and wastewater treatment can be significantly increased. 

It remains of great interest to find either appropriate material formulations or preparation methods, or both, that can make PVDF-based membranes more effective in eliminating the organic fouling problem but more cost-effective as well as easier to prepare, especially on a large scale, in spite of the fact that PVDF-based membranes have been produced and used for a few decades [10,11,12,13,14,15,16]. Extensive and intensive efforts have however been made to improve or enhance the anti-fouling performance of PVDF membranes used in water and wastewater treatment in recent years. One of the major focus areas in this line has been in the field of membrane material’s improvement or modification. This typically includes various approaches either in modifying existing membranes through surface grafting and surface coating, or in forming composite membrane materials through processes such as polymer or material blending, etc. Surface grafting [11,12] or surface coating [13,14,15] modification has been effectively and widely practiced. Its major challenge may be the difficulty in the control or retaining of the desired final membrane pore sizes and structures, and, perhaps, the higher additional cost in carrying out their scale-up applications. The blending method involves the mixing of one or more functional materials (or also called the additive) with the base membrane material to manufacture the membrane product directly [16,17,18]. The base membrane material usually provides the matrix structure and the additive provides the desired functionality of the membrane products. The blending approach is generally easier or more versatile to execute in engineering application, but the method may however face a compatibility issue between the base and additive membrane materials. It is crucial to select or synthesize the desired additive that can provide the needed compatibility with the base membrane material as well as the anticipated functionalities (especially multiple ones) of the obtained membrane products.

In the blending direction, various types of additives have been introduced or tested for PVDF membrane modification, including hydrophilic polymers, amphiphilic copolymers, and inorganic particles, etc. The choices of additives can incur various changes in the membrane’s property and structure, as well as affect the conditions for the membrane preparation. Many past attempts have explored relatively simpler additives, such as polyvinylpyrrolidone (PVP) [19,20], polyethylene glycol (PEG) [21], titanium dioxide (TiO_2_) [22,23], poly(styrene)-*g*-poly(ethylene glycol) methacrylate (PS-*g*-PEGMA) [24], and PVC-graft-poly(ethylene glycol) methyl ether methacrylate (PVC-*g*-P(PEGMA)) [25,26], and a summary of those studies can be found in Liu et al.’s review paper [3]. The predominant trend has focused on the hydrophilic polymer additives because they can endow surface hydrophilicity to the blend membranes fabricated from conventional hydrophobic base membrane materials, including PVDF. However, some studies have shown that the hydrophilic properties as well as the anti-fouling resistance of the obtained membranes from those simple additives may diminish or even ultimately disappear over time, as a result of dissolution, detachment, or leaching out from the prepared membranes, attributed to their poor compatibility with the base hydrophobic membrane materials [19,20,27,28]. To solve this issue, di-block amphiphilic copolymer additives that contain both hydrophobic and hydrophilic segments have been explored. The hydrophilic chains or segments of the additive are expected to appear on the surface of the membrane while the hydrophobic chains or segments of the additive stay with the base membrane material (due to their better similarity) to form the overall membrane’s structure [29]. Typical examples including poly(methyl methacrylate-*g*-polyoxyethylene methacrylate) (P(MMA-*g*-POEM)), poly(methyl methacrylate)-*g*-poly(ethylene glycol) methacrylate (P(MMA-*g*-PEGMA)), or poly(2-dimethylaminoethyl methacrylate)-block-poly(methyl methacrylate) (P(MMA-*b*-PDMAEMA)) were reported as the additives for PVDF membranes [30,31,32]. The methacrylate backbone (PMMA) has been considered well miscible with PVDF to obtain the long-term stability of the membrane with the surface enriched POEM, PEGMA, or PDMAEMA chains or segments endowing the modified PVDF membrane with expected hydrophilicity and thus fouling resistance. The modified PVDF membrane was shown to have a lower decline rate in the permeate flux during bovine serum albumin (BSA) solution filtration [30] or a lower adsorption of BSA when the membrane was immersed in BSA solution [32]. 

There has also been the interest to develop or enhance the oleophobic property of water filtration membranes in recent years [33,34,35,36]. This may be partly due to the challenge that most polymeric membranes available in the market are unable to be directly used for oil–water separation applications, as a result of the rapid and severe membrane fouling. On the other hand, it is envisaged that membranes with oleophobic property will most likely have better anti-fouling performance, especially for organic or biological fouling that is often irreversible [37,38]. This is due to the consideration that oils usually have higher adhesion tendency or strength than most other organic matters on an organic membrane surface [39,40]. Zhao et al. reported intrinsically hydrophilic foams can be modified to be oleophobic–hydrophilic through covalent grafting of fluorinated methacrylate only. The modified foams exhibit oil water larger than 140° [41]. In general, there have been relatively very limited researches available in the literature on a water filtration membrane prepared by the blending method for both hydrophilic and oleophobic surface properties [29,42]. Although most membranes’ anti-fouling strategies adopted the hydrophilic modification approach, Bai and co-workers had explicitly proposed to prepare a modified PVDF membrane with both hydrophilic as well as oleophobic surface property as a better solution for membrane anti-fouling performance [29]. They prepared a tri-block copolymer additive with the hydrophobic–hydrophilic–oleophobic structure to provide both the desired compatibility with PVDF as well as the hydrophilic and oleophobic property of the obtained PVDF membrane. Experimental results indeed showed that the modified PVDF membrane with both hydrophilic and oleophobic surface property performed better than the one with hydrophilic surface property only in the anti-fouling performance against some organic and oil pollutants [29]. However, their earlier work [29,33,42] did suffer from some drawbacks, such as that either they did not reach very high hydrophilicity or oleophobicity, or used some expensive or toxic materials or solvents in the preparation of the additives, or the used methods were very difficult for scale-up applications [38,42,43,44].

In the present work, we report the preparation of a new triple-component copolymer additive of P(St_x_-*co*-MAA_y_)-*g*-fPEG_z_ by using a relatively easier method as well as cheaper and more environmentally friendly materials. In the copolymer additive, a strong anchorage component of St- (styrene) can provide hydrophobic interaction with PVDF to ensure their compatibility, and the highly hydrophilic component of MAA (methacrylic acid) and the oleophobic segment of fPEG (perfluoroalkyl polyethylene glycol) in the flexible chains are expected to appear or enrich on the surface of the prepared membrane to provide the desired both hydrophilic and oleophobic surface properties. The obtained copolymer additive was blended with PVDF to fabricate modified PVDF membranes through the common non-solvent-induced phase separation method. Particularly, the different ratios of St to MAA and thus to fPEG in the copolymer additive were examined for their effect on the properties of the obtained PVDF membranes. The prepared membranes were characterized through various analyses and were also tested for the separation of a few types of typical organic pollutants in water solutions to evaluate the membranes’ anti-fouling performance.

## 2. Materials and Methods

### 2.1. Materials

The monomers of styrene (St, 99%, from Macklin, Shanghai, China) and methacrylic acid (MAA, 99%, from Aladdin, Shanghai, China) were first passed through an alumina column, respectively, to remove the inhibitors coming with the products, and then were stored at −20 °C prior to use in the study. 2, 2′-Azobisisobutyronitrile (AIBN, 98%, from Aladdin) was recrystallized from anhydrous ethanol before use. Dicyclohexylcarbodiimide (DCC, from Macklin), 4-dimethyaminopyridine (DMAP, from Macklin) and perfluoroalkyl polyethylene glycol (fPEG, from Du Pont, Wilmington, DE, USA) were used as received without further treatment. N, N-dimethyformamide (DMF, from Jiangsu Qiangsheng Chemical Co., Changshu, China) was dried by a 4A molecular sieve before use. Polyvinylidene fluoride (PVDF, FR904, supplied by Shanghai 3F New Material Co., Shanghai, China) was dried at 100 °C for 12 h before being used as the base material to prepare the membranes. Other materials, including N-methypl-2-pyrrolidone (NMP), polyvinylpyrrolidone (PVP, K30), bovine serum albumin (BSA), humic acid (HA, ≥90%), and n-hexadecane (≥98.5%) were supplied by Macklin Corp. Deionized water (DI, 18.2 MΩ cm) produced from a Millipore water purification system (Millipore ZRXQ015TO) was used as needed in the experiments. 

### 2.2. Synthesis and Characterization of the Triple-Component Copolymer Additive

**Scheme 1:**
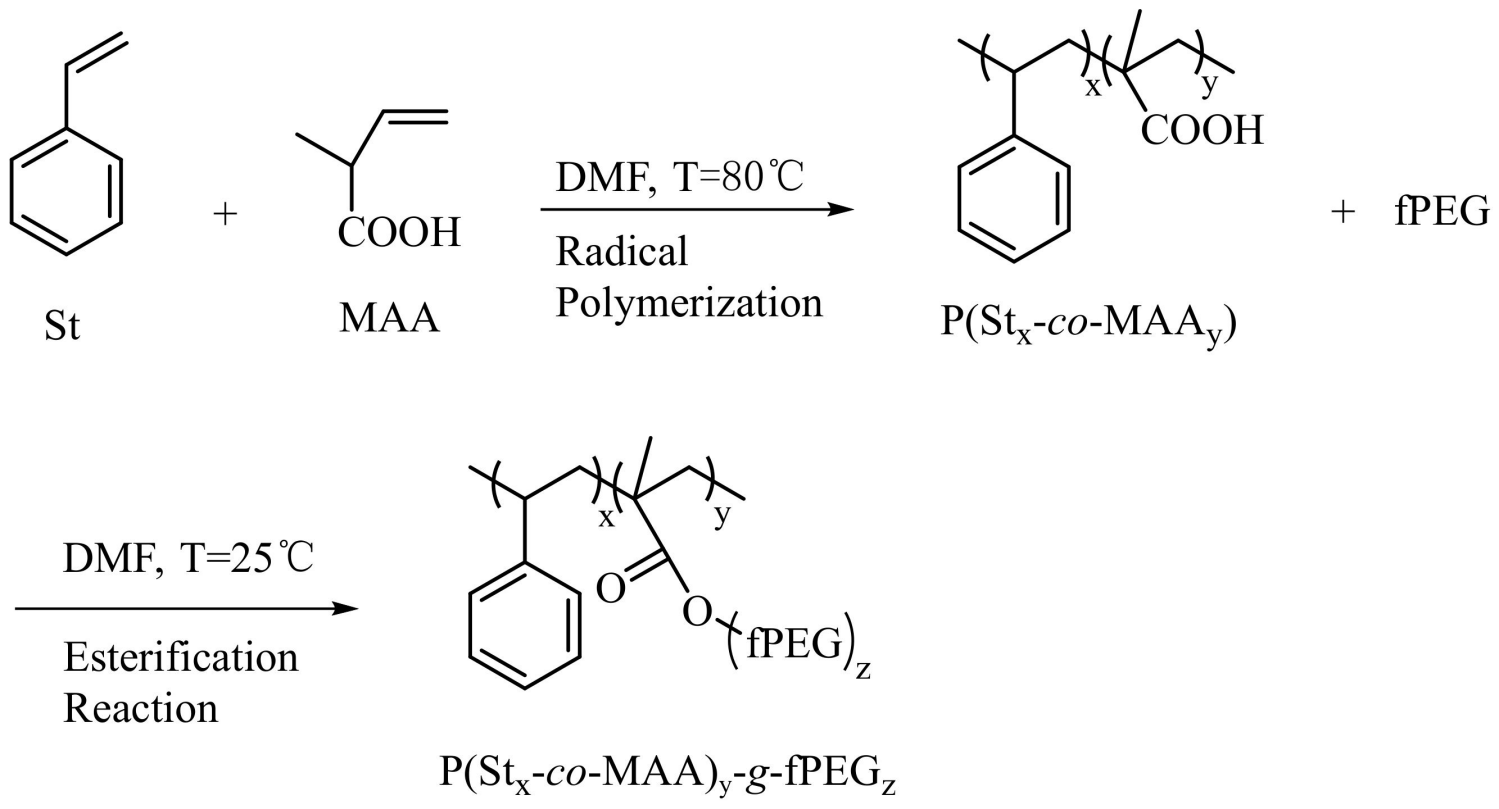
An example in the synthetic procedure and composition of the triple-component copolymer additive.

The copolymer additive of P(St_x_-*co*-MAA_y_)-*g*-fPEG_z_ was prepared through a two-step process consisting of a radical polymerization followed by an esterification reaction, as shown in Scheme 1 below.

In the first step, the St and MAA monomers were dissolved in anhydrous DMF solvent with AIBN added as the reaction initiator to obtain the dual-component copolymer of P(St-*co*-MAA) through the free radical polymerization method. To vary the hydrophilic (by MAA) and thus the oleophobic (by fPEG) property in the ultimate additive product, two different molar ratios of St:MAA at 1:1 and 5:1, respectively, were used. Specifically, 148.7 mL (1.3 mol) or 743.5 mL (6.5 mol) of St and 111.5 mL (1.3 mol) of MAA were added into 130 mL of DMF in a double-*g*lazing reactor. The contents in the reactor were pumped and mixed with nitrogen gas from a pressurized cylinder by a metering pump for two full displacement cycles to thoroughly remove any oxygen remaining in the reaction mixture. Then, the reactor was stirred at 300 rpm for 20 min under the room temperature (22–25 °C) to obtain a homogeneous solution. It was further deoxygenated with a steady supply of nitrogen gas bubbles injected at the bottom within the reactor for 20 min. After that, 6.4 g (0.039 mol) of AIBN was added into the solution in the reactor with continuous stirring. It was followed by another two full displacement cycles as mentioned earlier for the removal of any potential oxygen residual in the reactor. Subsequently, the mixture in the reactor was heated to 80 °C for the polymerization to continue for 11 h under a nitrogen atmosphere (with nitrogen blanket above the solution surface). Finally, the mixture in the reactor was first diluted by adding some acetone and then was discharged into a large basket of water with stirring for the synthesized copolymer to precipitate. The product was collected by filtering the solids in the liquid in the basket through a vacuum filtration system. The collected product was washed first by water and then by a mixture of water/ethanol at the ratio of 1:1 several times to fully remove any unreacted residuals of the St (if any) and MAA monomers. The obtained product, denoted as P(St_x_-*co*-MAA_y_), was finally dried with a freeze-dryer until a constant weight and was kept in a desiccator at room temperature for further use. 

In the second step, P(St_x_-*co*-MAA_y_) and fPEG were reacted through an esterification reaction to obtain the desired triple-component copolymer additive of P(St_x_-*co*-MAA_y_)-*g*-fPEG_z_. For example, P(St-*co*-MAA) (100 g) and fPEG (108 g) were first dissolved in anhydrous DMF (1.5 L) in the reactor under room temperature. Then, DMAP (4.08 g) was added into the solution in the reactor as a catalyst. Following that, DCC (0.5 L), dissolved in 0.5 L dehydrated DMF, was added dropwise into the reactor. The mixture in the reactor was stirred for the esterification reaction to continue for 7 days under the nitrogen blanket atmosphere. Finally, the contents in the reactor were discharged into a water/ethanol (1/1) mixture for product precipitation. The solids in the mixture were then collected through a vacuum filtration system. The collected product was washed several times with the water/ethanol mixture, and then freeze-dried. The final product is denoted as P(St_x_-*co*-MAA_y_)-*g*-fPEG_z_, where x, y, and z indicate the relative amounts of the different components in the copolymer additive.

To confirm the successful preparation of the intermediate copolymer of P(St_x_-*co*-MAA_y_) and the final additive copolymer of P(St_x_-*co*-MAA_y_)-*g*-fPEG_z_, the respective samples were analyzed by 1H-nuclear magnetic resonance (NMR). The 1H-NMR spectra were recorded on a Bruker Inova 400 MHz instrument, using dimethylsulfoxide-d6 as the solvent. Chemical shifts were given in parts per million from that of tetramethylsilane (TMS) as an internal reference.

### 2.3. Membrane Preparation

Flat membranes were prepared through the common non-solvent-induced phase separation method. Both the base (or control) PVDF membrane and the modified PVDF membranes were obtained at a total membrane polymer content of 18 wt% in the cast solution (5% PVP was also added as a porogen). The modified PVDF membranes had a material composition by blending the copolymer additive of P(St_x_-*co*-MAA_y_)-*g*-fPEG_z_ with PVDF at two different ratios (1:9 and 3:7), and with the obtained additive of P(St_x_-*co*-MAA_y_)-*g*-fPEG_z_ at two different x-to-y ratios (x:y = 4:1 or 1:1) (note: the 1H NMR analysis showed always with z = y, i.e., the esterification reaction was nearly fully achieved under the study conditions). NMP was used as the polymer solvent and water as the non-solvent. The procedures for the membrane preparation were similar to those described in the literature [27]. In brief, the membrane materials were dissolved in NMP and mechanically stirred in a glass container at 80 °C in an oil bath for 48 h to obtain a casting solution. After being filtered and then degassed under a vacuum, the cast solution was spread onto a clean glass plate with a Doctor Blade Film Applicator (Elcometer 3600) at a film thickness of 250 µm. The membrane film on the cast glass plate was then immersed into a DI water coagulation bath at 50 °C for 5 h for the phase inversion and surface segregation of the different polymer components in the membrane to occur. The film was then transferred into another DI water bath at room temperature for 2 days. Finally, the membrane was removed from the coagulation bath, dried in air at room temperature for 2 days, and then stored in a desiccator for further use. Five types of membranes were prepared in this study. Table 1 shows the relevant conditions in the preparation of these membranes and, also, some of their measured membrane properties. M0 is the control PVDF membrane and M1 to M4 are the modified PVDF membranes with two different PVDF-to-additive ratios and using two different compositions of St versus MAA in the additive. 

### 2.4. Characterization of Prepared Membranes

A scanning electron microscope (SEM, Phenom Pro) was used to examine the cross-section structures and the surface morphologies of the membranes prepared under different conditions. For cross-section observation, a membrane sample was freeze-fractured in liquid nitrogen to obtain a fresh surface. Membrane samples were coated with gold particles before the SEM analysis. From the surface images, a nominal surface pore size for each type of the prepared membrane samples was also estimated by the software of “Nano Measure”. For every sample, randomly 100 pores were measured and averaged. 

A Fourier transform infrared (ATR-FTIR) spectrometer (Thermo Nicolet 6700) was used to verify the presence of various functional groups in the membranes. The samples were placed on a diamond crystal and the spectrum was obtained between 500 and 4000 cm^−1^ for 32 scans at a resolution of 4 cm^−1^, following the standard analysis procedure of the equipment.

The mechanic properties of the prepared membranes were measured by Instron equipment (Model 5944). A dyed membrane was cut into pieces of 1 cm × 10 cm and then vertically attached to both end of the instrument. The dragging rate of the grip was set at 1 cm/min in the tests. The tensile strength was reported by the instrument when the two fixtures moved until the membrane fractured. At least six tests for each type of the membranes were measured and the average value were reported.

Contact angle (CA) measurements with water and oil (hexadecane) for the prepared membranes were conducted to evaluate their hydrophilic and oleophobic characteristics. The measurement was performed at room temperature by a contact angle goniometer (Ramé-hart 500). For the water contact angle (WCA), a membrane sample was fixed on the sample stand of the instrument in a horizontal position, with the target membrane surface facing up. A water droplet (3 µL) was injected slowly from a micro-syringe onto the membrane surface. The water-drop image on the membrane surface was captured and analyzed by the instrument to give the WCA value. For the oil contact angle (OCA) of the membrane-in-water (simulating the filtration situation), the membrane sample was immersed in the measuring cell filled with DI water and fixed at a horizontal position with the target membrane surface facing down in the cell. An n-hexadecane droplet (3 μL) was carefully injected onto the membrane surface and the OCA was measured by the instrument. For all the membrane samples, each of the WCA or OCA measurements was made at eight different locations on the membrane surface and an average value of those measurements was reported as the representative one in this study.

The porosities of the prepared membranes were estimated by the liquid wetting method. Firstly, the thickness (δ, m) of a membrane sample was obtained directly from the digitized SEM image. The membrane sample was cut into the area size (A) of 4 × 4 cm^2^ and then immersed in a water/ethanol (1:1) mixture. The membrane was completely wetted for 24 h. Then, the wet membrane was taken out, had surface water removed by a tissue paper, and was then weighed (W1, g). The wet sample was then vacuum dried to a constant weight and it was weighed again (W2, g). The weights of the wetted and dried membrane samples were obtained from an electronic scale (Statorius, Guis). The porosity (*ε*, %) of the membrane sample was estimated by Equation (1) as follows:

(1) $$\varepsilon =\frac{W1-W2}{\rho \times A\times \delta }\times 100\%$$

where *ρ* (kg/m^3^) was the density of the water/ethanol mixture.

### 2.5. Protein Adsorption Test

The adsorption of protein to a membrane surface is often a concern in membrane anti-fouling performance because many organic membranes suffer from severe protein fouling. In this study, protein adsorption to the membrane surface was investigated with the typical protein of BSA. A membrane sample (cut in the size of 3 × 3 cm^2^) was first rinsed by phosphate buffered solution (PBS, pH = 7.4) to remove any impurities that may adhere on the surface of the membrane. Then, the membrane was immersed into 50 mL of a BSA solution with the concentration in the range of 800–2000 mg/L in a flask. The contents in the flask were stirred in a shaking water bath (OLS Grant 200) at room temperature for 24 h to allow protein adsorption on the membrane sample to sufficiently reach the adsorption equilibrium. The concentrations of BSA in the solutions before and after the adsorption test were analyzed with a UV-vis (Ultraviolet-visible) spectrophotometer (Shimadzu UV3600, Kyoto, Japan) at 280 nm (the reading was converted to the weight concentrations through a calibration curve). The amounts of BSA adsorbed on the membrane samples were determined through a mass balance analysis.

### 2.6. Filtration Experiments

Experiments to determine the water permeation flux and anti-fouling properties of the prepared membranes were performed using an Amicon 8400 stirred dead-end filtration cell (Millipore) with a feed cell volume of 400 mL and an effective membrane filtration area of 41.8 cm^2^. The cell was filled with water or a test solution and stirred at 300 rpm with a magnetic stirrer to minimize the effect of concentration polarization. A constant filtration pressure (1 bar) was maintained by a pressurized nitrogen gas cylinder. Filtration was conducted under room temperature and the permeate from the membrane was collected within a desired time interval and weighed to determine the membrane’s permeation flux. 

The filtration operation was conducted in three stages. First, DI water was passed through the membrane at a pressure of 0.1 MPa until the flux became stable and continued for at least half an hour. The stabilized water flux was determined and denoted as *J*_0_; in the second stage, the cell was emptied and then filled with a model foulant solution to be tested. The filtration experiment was resumed under the same operational pressure as in the first stage and the permeate flux changes were monitored versus the filtration time for 2 h. The final flux was recorded and denoted as *J_p_*. In the third stage, the membrane was taken out from the cell for cleaning. Then, the cleaned membrane was put back into the cell. The filtration was conducted again with DI water under the same feed pressure as before and continued until the water flux was stabilized for 0.5 h. The stabilized flux in the third stage was denoted as *J*_1_ (the cleaning of the membrane at the end of the second stage was conducted by putting the membrane in DI water in a beaker under ultrasonic vibration at the frequency of 40 kHz for 10 min). The flux of the membranes *J*_0_, *J_p_*, *J*_1_ was calculated as seen in Equations (2)–(4), respectively.

(2) $$J_{0}=\frac{V_{0}}{A\Delta t}$$

(3) $$J_{p}=\frac{V_{p}}{A\Delta t}$$

(4) $$J_{1}=\frac{V_{1}}{A\Delta t}$$

where *V*_0_ (L), *V_p_* (L), and *V*_1_ (L) are the permeate volume, A (m^2^) represents the effective filtration area (41.8 × 10^−4^), and Δt (h) is the record time. 

Three types of foulant solutions made from protein (BSA), nature organic matter (HA), and oil (n-hexadecane) were used in the filtration experiments in the second stage to evaluate the anti-fouling performance of the prepared membranes for these typical and representative organic foulants. The feed concentration of BSA or HA solution was at 1 g/L, obtained by dissolving BSA or HA in PBS-buffered solution (pH = 7.4), respectively. The feed of the n-hexadecane-in-water emulsion had an oil concentration at 100 mg/L, prepared by dispersing hexadecane in DW with a homogenizer (Cole-Parmer, Labgen 700) at 10,000 rpm for 20 min.

The concentrations in the feed (*c_f_*) and permeate (*c_p_*) for BSA and HA were determined with a UV-vis spectrophotometer (Shimadzu, UV-3600) at the wavelengths of 278 and 400 nm, respectively, and those for the n-hexadecane were estimated with a TOC analyzer (Shimadzu, TOC-L CPH).

From the filtration experiments, the relative flux decay (RFD), the relative flux recovery (RFR), and the retention or removal rate (R) of the membranes were calculated as seen in Equations (5)–(7), respectively. These results are used to indicate the membrane’s anti-fouling and separation performance in this study.

(5) $$\mathrm{RFD}=\frac{J_{0}-J_{p}}{J_{0}}\times 100\%$$

(6) $$\mathrm{RFR}=\frac{J_{1}}{J_{0}}\times 100\%$$

(7) $$R=\frac{1-c_{p}}{c_{f}}\times 100\%$$

## 3. Results and Discussion

### 3.1. Characteristics of the Triple-Component Copolymer Additives

**Figure 1:**
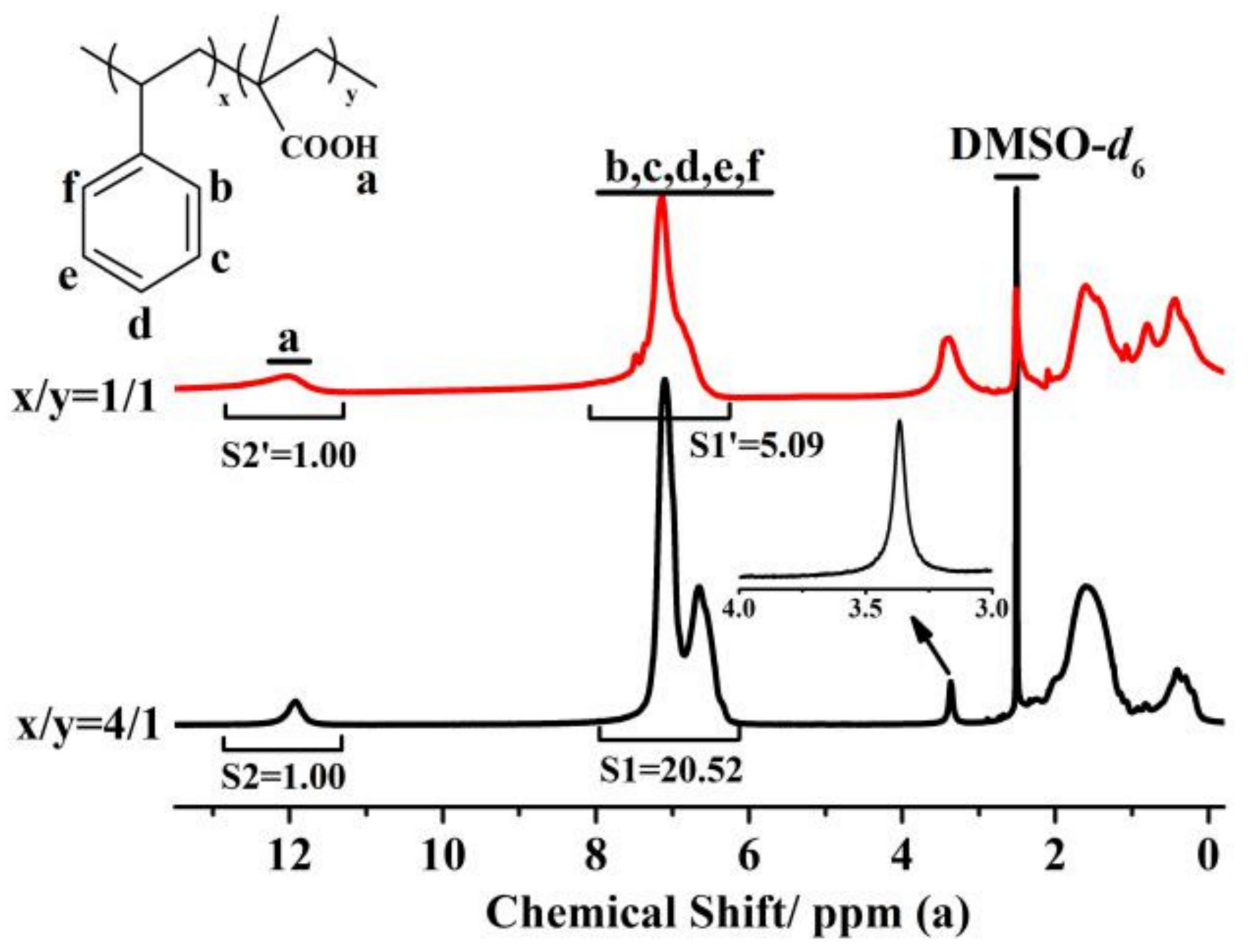

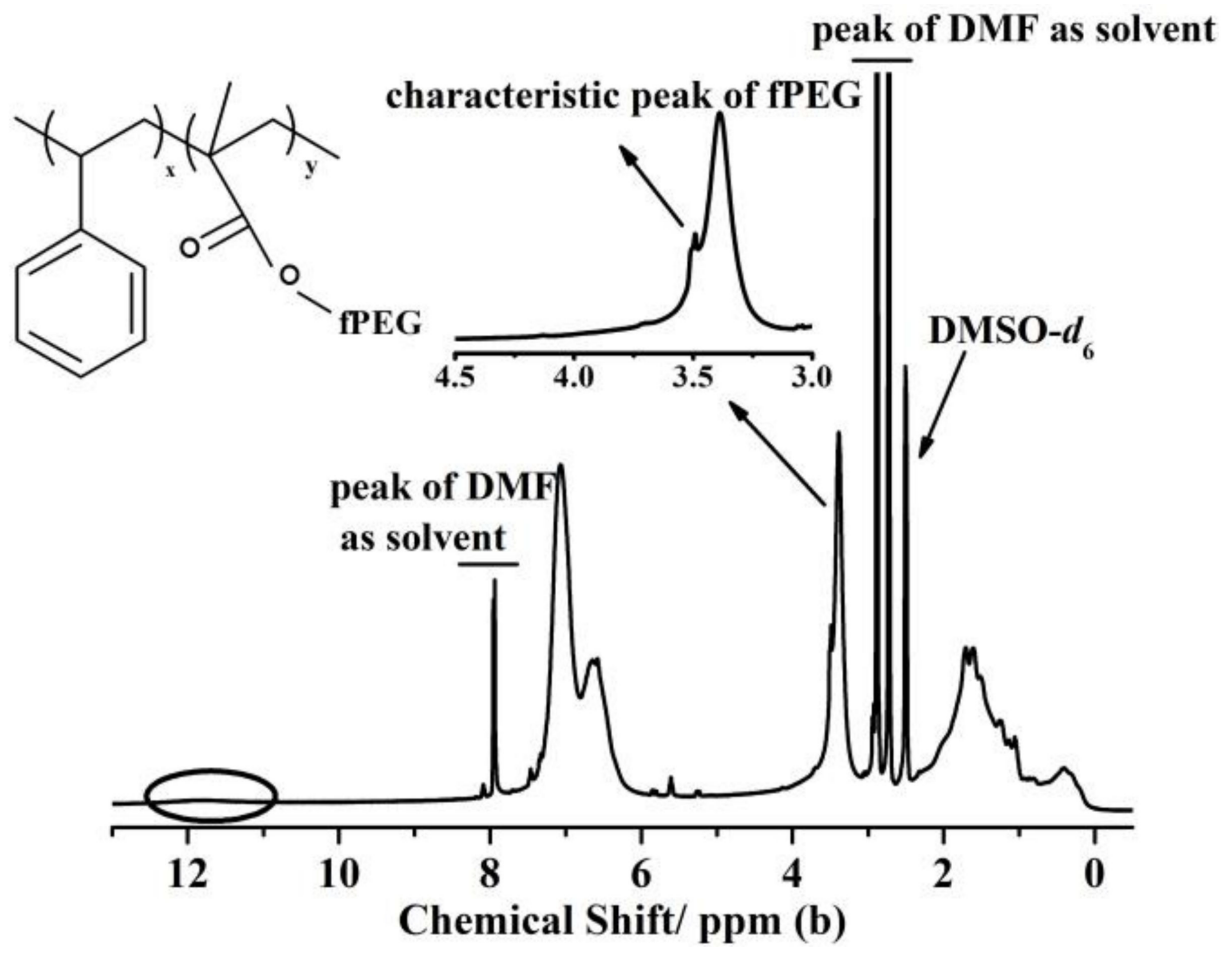
Typical ^1^H NMR spectra of the copolymers of (**a**) P(St_x_-*co*-MAA_y_) (with x:y = 4:1 and 1:1), and (**b**) P(St_x_-*co*-MAA_y_)-*g*-fPEG_y_ (with x:y = 4:1).

As shown in Scheme 1, the first step is to obtain the dual-component copolymer intermediate of P(St_x_-*co*-MAA_y_) from the two monomers of St and MAA via a free radical polymerization reaction. To confirm the successful copolymerization for P(St_x_-*co*-MAA_y_), ^1^H NMR spectra were obtained for the various samples. Figure 1a shows the typical ^1^H NMR spectrum of P(St_x_-*co*-MAA_y_) sample obtained with St:MAA ratio at 4:1 or 1:1 in the reaction. It can be identified that the typical signal of the carbonyl proton appeared at the location of 12 ppm in the ^1^H NMR spectrum, and the multiple peaks at the location of 6–8 ppm can be assigned to the five protons of the benzene ring in St [45]. Therefore, the spectrum in Figure 1a does support the dual-component structure of the expected P(St_x_-*co*-MAA_y_) intermediate. From the ^1^H NMR spectra (Figure 1a), the ratio of St:MAA (or x:y) in the P(St_x_-*co*-MAA_y_) intermediate can be calculated from the integration ratio of the styryl protons of the St repeating units (b, c, d, e, f, 6.10–7.95 ppm, 5H, integration value = *S*1 or *S*1′) and the carboxyl proton of the MAA repeating units (a, 11.31–12.81 ppm, 1H, integration value = *S*2 or *S*2′) with the following Equation (8):

(8) $$\frac{\left[St\right]}{\left[MAA\right]}=\frac{x}{y}=\frac{S1}{5S2}or\frac{S1\prime }{5S2\prime }$$

The results revealed that the P(St_x_-*co*-MAA_y_) intermediates obtained in the reaction with the St:MAA ratio at 5:1 or 1:1 led to the product with the x and y values at x:y = 4:1 and x:y = 1:1, suggesting that the synthesis reaction given in the first step in Scheme 1 by the free radical polymerization was indeed successful and effective under the test conditions. 

The triple-component copolymer additive of P(St_x_-*co*-MAA_y_)-*g*-fPEG_z_ was further prepared from P(St_x_-*co*-MAA_y_) and fPEG through an esterification reaction between the carboxyl and hydroxyl groups. The typical ^1^H NMR spectrum of P(St_x_-*co*-MAA_y_)-*g*-fPEG_z_ from the analysis is shown in Figure 1b. It can be found that a new and characteristic peak at 3.5 ppm [46], attributed to the perfluoroalkyl PEG (fPEG), appeared, suggesting the existence of fPEG segments in the obtained additive compound. It can also be noted in the ^1^H NMR spectrum that the characteristic signal located at 12 ppm for the carbonyl proton disappeared completely after the esterification reaction, indicating the complete esterification reaction of MAA with fPEG, or a complete conversion of P(St_x_-*co*-MAA_y_) to P(St_x_-*co*-MAA_y_)-*g*-fPEG_z_ in this case, with the values of z and y probably being the same. The obtained copolymer additives of P(St_x_-*co*-MAA_y_)-*g*-fPEG_y_ were found to be insoluble in water and only dissolved in strong polar solvents, such as NMP, DMF, and DMSO, etc.

### 3.2. Morphologies and Mechanical Strength of Prepared Membranes

**Figure 2:**
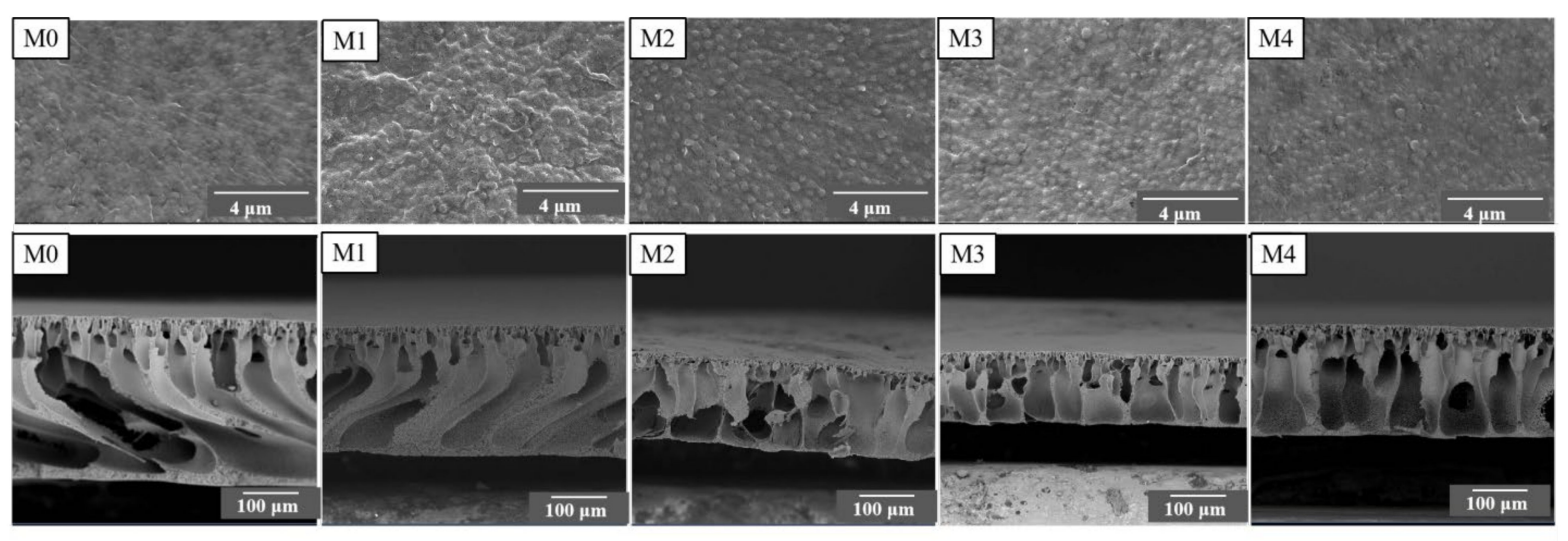
SEM images for the surface (upper row) and cross-section (low row) morphologies of the PVDF blend membranes M0 (weight ratio of PVDF: Additive = 10:0), M1 (weight ratio of PVDF: P(St_x_-*co*-MAA_y_)-*g*-fPEG_z_ (x:y = 4:1) = 9:1), M2 (weight ratio of PVDF: P(St_x_-*co*-MAA_y_)-*g*-fPEG_z_ (x:y = 4:1) = 7:3), M3 (weight ratio of PVDF: P(St_x_-*co*-MAA_y_)-*g*-fPEG_z_ (x:y = 4:1) = 9:1), M4 (weight ratio of PVDF: P(St_x_-*co*-MAA_y_)-*g*-fPEG_z_ (x:y = 4:1) = 7:3).

The morphology of the prepared membranes can be affected due to the blending of the copolymer additive with PVDF, resulting from the different compatibility among the base membrane polymer and the various segments of the additive polymer. Figure 2 shows the SEM images for the surfaces and cross-sections of the five types of membranes prepared under different compositions as listed in Table 1. For each membrane (from M0 to M4), the upper row shows the surface morphology and lower row the cross-section morphology. As expected, the PVDF membrane without the additive (i.e., M0) was generally less porous. With the addition of the additive polymer, the membranes became more porous (e.g., M1–M4 compared to M0). This can be attributed to the different polymer compatibility between the additive and PVDF (for M1 to M4) in comparison with that among PVDF itself (for M0). It was found that both the content of the additive in the membrane materials and the contents of the hydrophilic and oleophobic segments in the additive had a great effect on the morphology of the prepared blend membranes. At a lower hydrophilic and oleophobic content, i.e., x:y = 4:1 in P(St_x_-*co*-MAA_y_)-*g*-fPEG_y_ for M1 and M2, the increase in the ratio of additive:PVDF from 1:9 (for M1) to 3:7 (for M2) resulted in the membrane being more porous and yet still having a uniform structure. At a higher hydrophilic and oleophobic content, i.e., x:y = 1:1 for M3 and M4, however, the increase in the additive:PVDF ratio from 1:9 for M3 to 3:7 for M4 was found to make the prepared membrane M4 become more non-uniform in its structure. This suggests that the compatibility of the additive with PVDF was reduced and the dispersion of the additive in the PVDF base polymer became poorer. On the other hand, at the same lower ratio of additive:PVDF at 1:9, the M3 membrane, which contained the additive with a higher hydrophilic and oleophobic content (i.e., x:y = 1:1), as compared to the M1 membrane that contained the additive with a lower hydrophilic and oleophobic content (i.e., x:y = 4:1), was more porous and had better anisotropic structure. It seems clear that a balance may need to be sought between the ratio of additive added with PVDF and in the hydrophilic and oleophobic content in the additive to be used in order to achieve the desired or optimum material compatibility and membrane structure. The present experimental results appear to suggest the ratio of additive:PVDF at 1:9 with the additive having the ratio of x:y = 1:1 to be a good choice (e.g., the membrane M3). The experimentally measured porosity values for the five types of membranes (M0 to M4) are also included in Table 1, which shows a consistent trend of increase (except for M4 that had a lower porosity than M3, possibly due to a poorer dispersity between PVDF and the additive). The measured average surface pore sizes from the SEM images for the prepared membranes also showed the same trend of changes as that of the porosity; see Table 1.

The mechanical properties of the prepared membranes are often estimated in terms of the tensile strength and breaking elongation and the result are recorded in Table 1. From the result of tensile strength, the PVDF membrane M0 showed the highest value of 1.58 MPa. With the addition of P(St_x_-*co*-MAA_y_)-*g*-fPEG_z_, the tensile strength of the modified membranes appears to be reduced, with M1 at 1.52, M2 at 0.98 and M3 at 1.08, respectively. M4 showed the lowest tensile strength of 0.67 MPa. The breaking elongation also decreased, with 57.10% for M0, 56.03% for M1, 19.11% for M2, 33.94% for M3, and 19.46% for M4. The results indicate that the lower compatibility between PVDF and P(St_x_-*co*-MAA_y_)-*g*-fPEG_z_ decreased the mechanical strength of the PVDF/copolymers composite membranes to a certain extent as compared to the solo PVDF membrane. As intended for low operation pressure of MF or UF membranes, the tensile strength of the composite membranes is generally acceptable.

### 3.3. Surface Composition of Prepared Membranes

**Figure 3:**
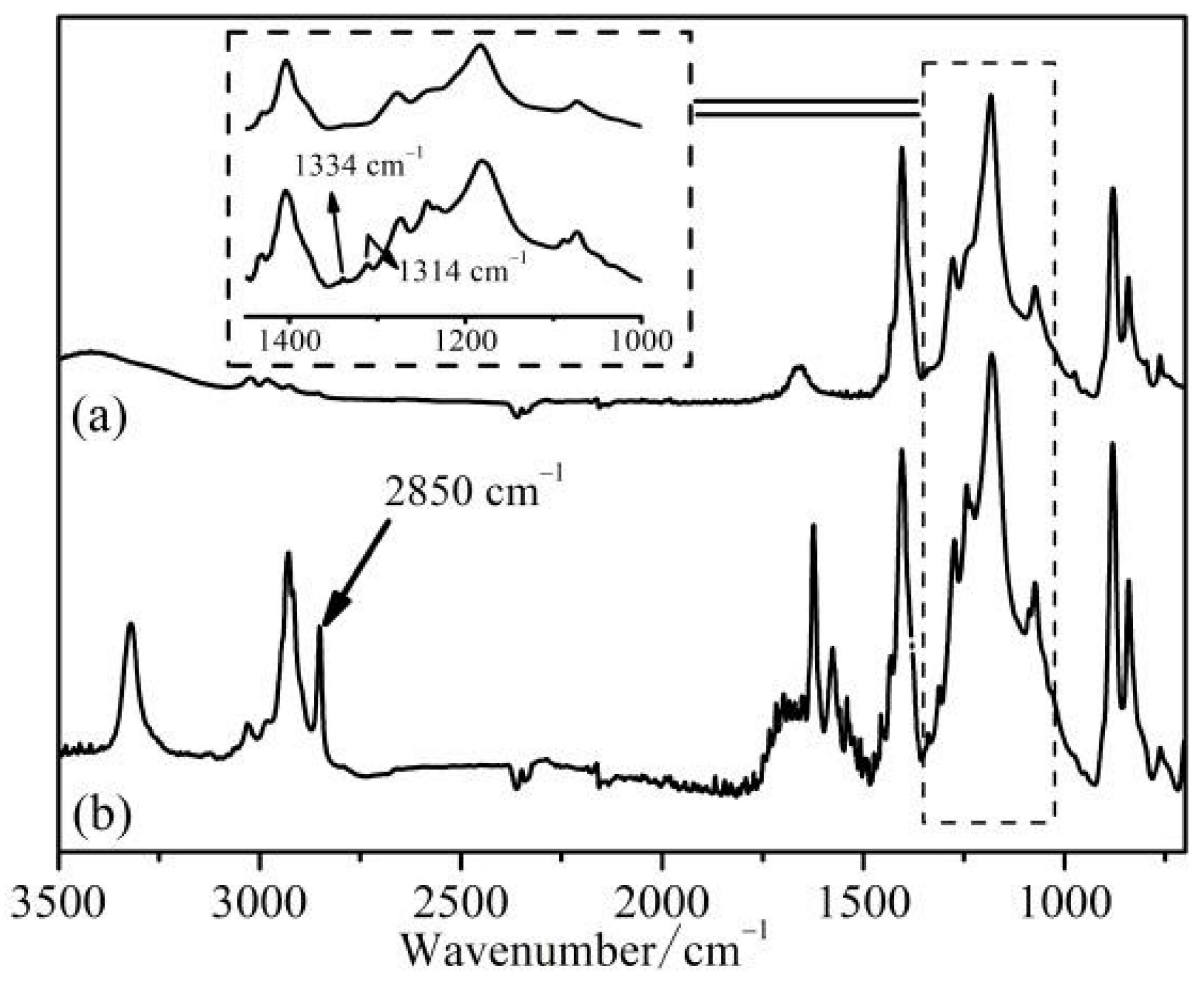
ATR-FTIR spectra of PVDF membrane M0 (**a**), and the modified PVDF membrane M3 (**b**) with the additive of P(St_x_-*co*-MAA_y_)-*g*-fPEG_y_ at x:y = 1:1.

The triple-component copolymer additive of P(St_x_-*co*-MAA_y_)-*g*-fPEG_z_ is expected to achieve three functions: the St component provides good compatibility with PVDF and would root in the membrane structure, and the MAA and fPEG components will desirably appear or enrich on the surface of the modified PVDF membrane (due to their poor compatibility with PVDF during the non-solvent-induced phase separation process) and thus provide the near-surface hydrophilicity by MAA and the forefront-surface oleophobicity by fPEG to the obtained membranes. The additive, to some extent, may also act as a porogen to affect the porous structure of the membranes. To confirm whether the hydrophilic and oleophobic segments of the copolymer additive were indeed present on or migrated to the surface of the obtained membranes during the membrane formation process [30], the ATR-FTIR spectra for the PVDF membrane M0 and the modified PVDF membrane M3 were collected. As shown in Figure 3a, the characteristic absorption band for CF_2_ of the PVDF membrane (M0) appeared at the region of 1120–1280 cm^−1^ [47]. A few new peaks apparently appeared in the spectrum of the modified PVDF membrane (M3); see Figure 3b. The peak at 1624 cm^−1^ can be assigned to the C = O stretching vibration of the ester groups, which indicated the successful esterification reaction between P(St_x_-*co*-MAA_y_) and fPEG. The absorption band at about 1314 cm^−1^ is known to belong to the C-H stretching of the CH_2_–O groups in the fPEG chains and that at about 2850 cm^−1^ can be assigned to the C–F stretching vibration from fPEG. In addition, the small peak at 1334 cm^−1^ could be considered to come from the C–F stretching vibration of the –CF_3_ groups in the additive [38,48]. Therefore, the FTIR results in Figure 3 do provide evidence on the appearance or migration of the additive components, particularly the oleophobic perfluoroalkyl segments of fPEG, on the surface of the modified PVDF membranes.

Thus, by fine-tuning the non-solvent-induced phase separation process, it may be possible to greatly enhance the migration and enrichment of the hydrophilic segment (MAA) and oleophobic segment (fPEG) of the additive on the prepared membrane surface, which would form another research area for further study.

A further XPS analysis (ESCALAB 250 XL, Thermo Fisher Scientific) for the surfaces of the various membranes was also conducted and the peaks of the curve-fitted C1s core-level spectrum for each membrane were resolved into peaks representing different chemical environments, using a sum of Lorentzian–Gaussian functions, from low to high binding energy (BE) for C–C (around 284.8 eV), C–H (around 286.1 eV), C–O (around 288.1 eV), C–F_2_ (around 290.7 eV), and C–F_3_ (around 291.6 eV) [49]. The obtained results are given in Table 2.

The presence of C–O on the PVDF (M0) membrane may be considered entirely from the presence of PVP added in the cast solution during membrane preparation. The increased amount of C–O on the modified PVDF membranes (M1–M4) can be considered to mainly result from the MAA segment in the additive. In other words, the hydrophilicity of the modified membranes is greatly enhanced due to the presence or enrichment of MAA segments on the membrane surfaces. As expected, the PVDF (M0) membrane did not show any C–F_3_ groups on its surface, but the modified PVDF (M1–M4) membranes obviously showed this functional groups on their surfaces that can only come from the fPEG segments in the additive. The surface presence or enrichment of the C–F_3_ groups would lead to the desired oleophobicity of the developed membranes for possible fouling-release property or potential. In addition, the XPS results in Table 2 also appear to indicate that the C–F_3_ groups on the surface became more significant with the increase in the additive ratio. Although M4 had the highest amount of the C–F_3_ groups on the surface, the membrane was found to have poorer compatibility between the additive and PVDF materials, which may favor the C–F_3_ groups to migrate or enrich to the surface during the phase separation stage in the membrane preparation process.

### 3.4. Membrane Surface Wetting Properties

The appearance and enrichment of the MAA and fPEG segments on the modified membranes’ surface to provide the desired hydrophilic and oleophobic property may be further verified from the surface wetting properties of the prepared membranes. The values of the static WCA and underwater OCA are used to indicate the hydrophilicity and oleophobicity of the prepared membranes for water treatment applications. A stronger hydrophilicity of the membrane surface would usually have a lower WCA value while a stronger oleophobicity of the membrane surface would usually show a higher OCA value. The measured results for the WCA and the OCA of the five types of prepared membranes are also included in Table 1. It is found that the unmodified PVDF membrane, M0, was typically more hydrophobic, with the highest measured WCA of 79.33°. By adding the synthesized triple-component copolymer additive into PVDF, the modified PVDF membranes generally had greatly reduced WCA values (around 60° or much less). The results hence support that the surface hydrophilicity of the modified PVDF membranes was enhanced, due to the presence and, probably, the enrichment of the hydrophilic segment of MAA from the blended P(St_x_-*co*-MAA_y_)-*g*-fPEG_z_ additive. 

The hydrophilicity of the modified PVDF membranes also appeared to depend on the ratio of PVDF to the additive as well as the content of the hydrophilic segment (MAA) and oleophobic segment (fPEG) in the additive, with a complicated but similar effect, as discussed earlier, to the membrane’s morphology. For example, membrane M3 that had a blending ratio of PVDF:additive at 9:1, with high hydrophilic and oleophobic content of x:y = 1:1 in the additive, showed the lowest WCA value of 48.8° (more than 30° less than that of M0), suggesting its strongest surface hydrophilicity. However, if the blending ratio became too high, the high hydrophilic and oleophobic content from the additive may cause its poor dispersity and thus affect the uniformity of the prepared membrane surface, which can compromise the effect of the additive, such as in the case of M4 whose WCA became higher (less hydrophilic) than that of M3. 

**Figure 4:**
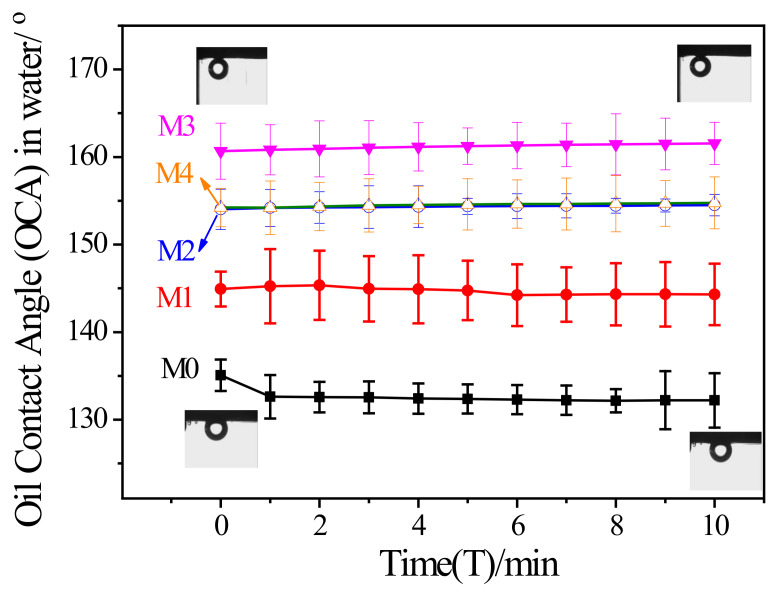
Measured OCA for the various PVDF-based membranes prepared.

It is interesting to note that the modified PVDF membranes also became much more oleophobic in water. As can be observed in Figure 4 (or found in Table 1), the OCA for the various membranes was around 135° for M0, 145° for M1, 154° for M2 and M4, and 160° for M3, respectively. Compared to 135° for the PVDF membrane M0, the significantly increased OCA for all the modified PVDF membranes indicated their enhanced oleophobicity. Particularly, M2, M3, and M4 may be even considered as super-oleophobic in water (OCA > 150°). It was found that the oil droplet on the various modified PVDF membranes (M1 to M4) during the OCA measurement was very stable during the 10 min observation, except for M0, which had a small decrease initially (see Figure 4). Noteworthily, the M3 membrane again showed the highest under-water oleophobicity. The enhanced oleophobicity of the modified PVDF membranes to a large extent may be attributed to the presence or enrichment of the oleophobic segment from the copolymer additive on the surfaces of the obtained membranes.

Both the significantly enhanced hydrophilic as well as oleophobic properties of the modified PVDF membranes (even at a relatively small ratio of the additive added) provide evidence that the hydrophilic and oleophobic segments of the copolymer additive may be indeed enriched on the membrane surface during the membrane-forming or phase inversion process. Considering the large difference in the surface energy of MAA (much higher) and fPEG (much lower) with that of St and PVDF, one may expect that MAA and fPEG are much less compatible with St and PVDF. As a result, St and PVDF stayed together and formed the membrane matrix structure while MAA and fPEG tended to migrate to the surfaces of the membranes. The benefit of the special wetting behavior of the obtained membranes may be further explained. In an oil-in-water system, due to the hydrophilic nature, a water layer can instantly be formed directly on the membrane surface. When an oil droplet approaches the membrane, the outer oleophobic terminal segment of fPEG groups from the additive will reject it, and, at the same time, the surface water layer can also prevent the oil droplet from direct contact with the membrane surface [50,51].

In the literature, although there was reporting on a modified PVDF membrane with underwater OCA at 157°, obtained through complex construction of multi-scale surface structure [52]_,_ there have not been any reports on modified PVDF membrane achieved underwater OCA at as high as 160°, obtained by the polymer blending method and directly through the non-solvent-induced phase separation process.

From the fact that the M3 membrane was both more hydrophilic and more oleophobic than M1, M2, and M4, one can conclude that a proper ratio of the hydrophilic (MAA) and hydrophobic (St) segments in the additive and a proper blending ratio of the additive with PVDF would have a great impact on the performance of the prepared membranes. An optimization in this aspect may be of importance for further study.

### 3.5. Adsorption to BSA Protein

Protein fouling is often a common and challenging problem with conventional membranes for many separation applications and it is always desirable to eliminate this effect as much as possible. Protein fouling is usually caused by the adsorption of protein onto the external and internal surfaces of a membrane, especially those made from polymeric ones, such as PVDF, that are highly hydrophobic. To evaluate the anti-fouling performance for protein, the prepared various PVDF-based membranes were placed into a BSA solution to examine the extent to which the BSA protein may be adsorbed onto these membranes. 

**Figure 5:**
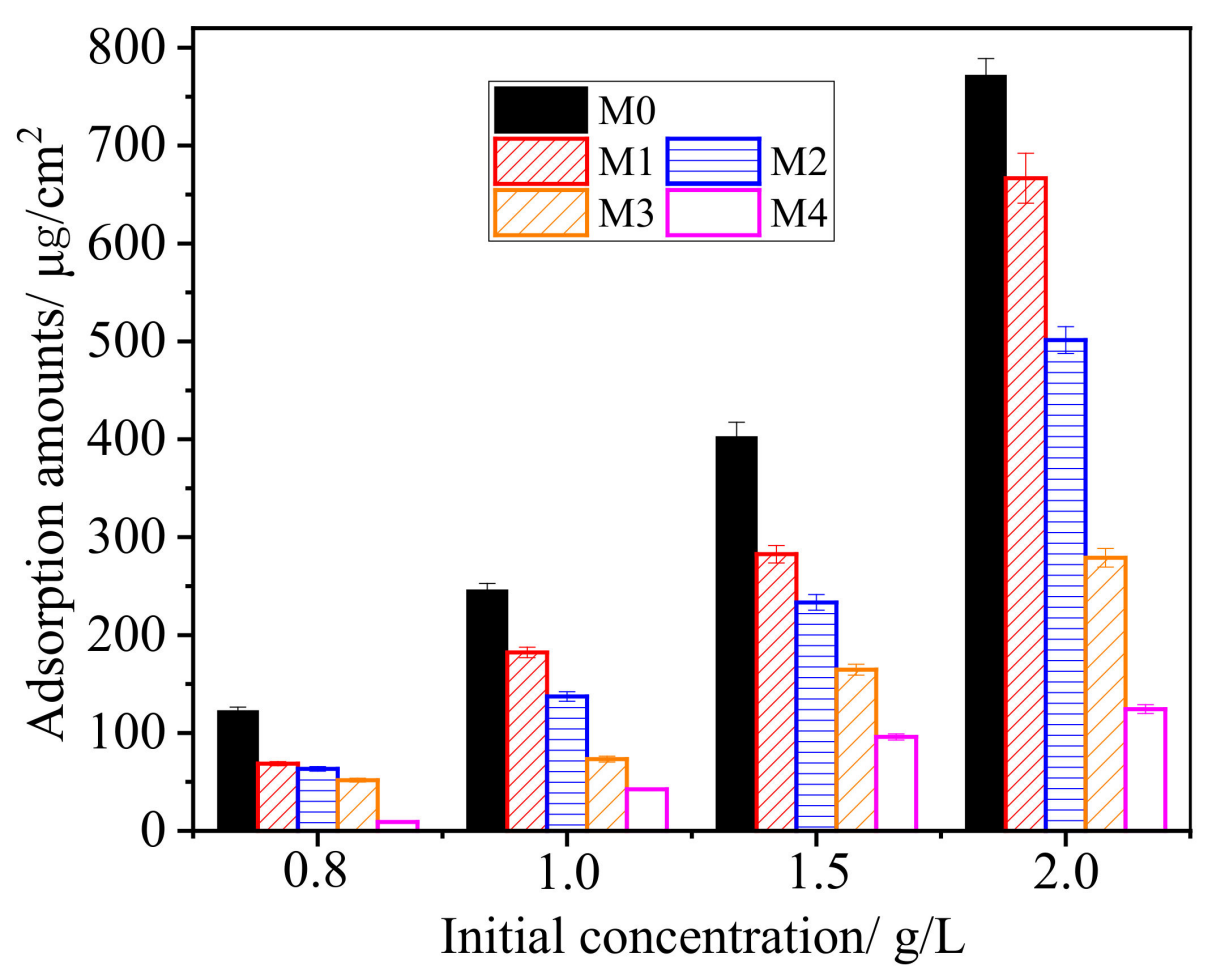
Adsorption amounts of BSA on various prepared PVDF membranes under different BSA concentrations in the solutions.

Figure 5 shows the adsorption amounts of BSA on the various types of membranes under different BSA concentrations. Although the adsorption amount for all the membranes showed an increase with the BSA concentration, the modified PVDF membranes with the copolymer additive of P(St_x_-*co*-MAA_y_)-*g*-fPEG_z_ greatly reduced the amount of BSA adsorbed. The trend in the adsorbed protein amounts seemed to be consistent with both the increasing in the hydrophilicity (decreasing in WCA) and oleophobicity (increasing in underwater OCA) of these membranes. For instance, at the initial BSA concentration of 0.8 g/L, M0 had the highest BSA adsorption of 122.2 μg-BSA/cm^2^-membrane. In contrast, the BSA adsorption amount on the M3 membrane was the lowest and at only about 7% of that for M0. At the high BSA concentration of 2 g/L, the adsorption on M3 was also only about 16% of that on the M0 membrane, and was also the lowest of all the membrane samples tested. The improved anti-fouling performance to BSA adsorption for the modified membranes may be explained as follows. The hydrophilic segments of the additive can appear or enrich on the membrane surface. Then, hydrogen bonds were formed between the hydrophilic groups of the additive and the water molecules in the solution, which induced the formation of a hydration layer on the membrane surface. That can sterically inhibit the close contact of protein to membrane surfaces for adsorption to take place [53,54].

### 3.6. Anti-Organic Fouling Performance during Filtration Separation Tests

**Figure 6:**
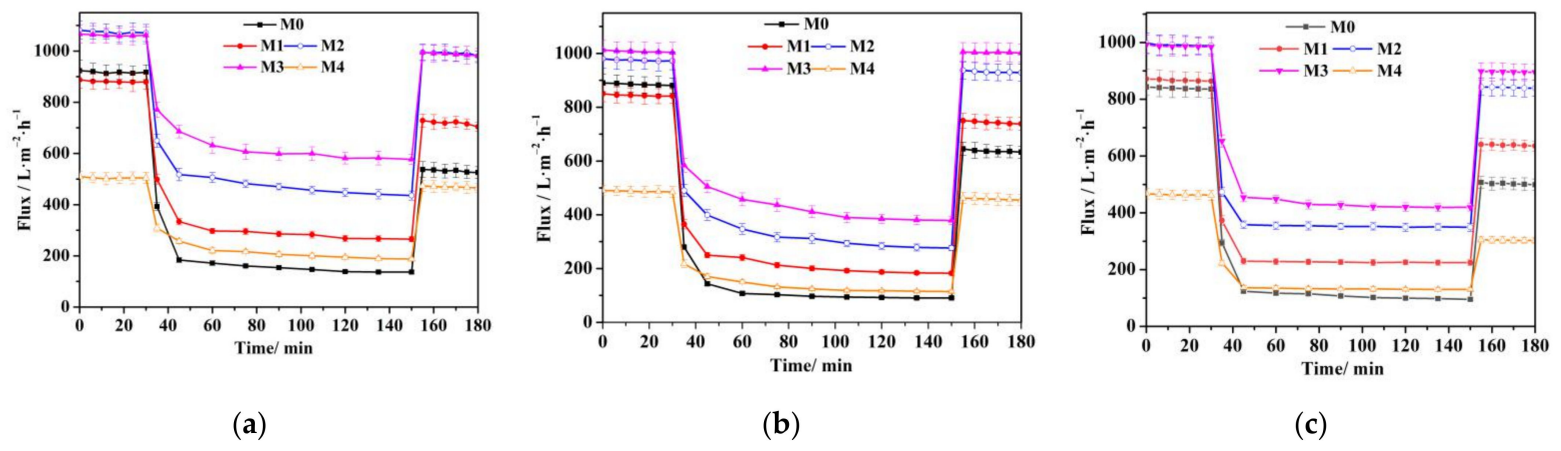
Water flux changes with filtration time for the various prepared membranes during the filtration of (**a**) BSA (c_f_ = 1000 mg/L), (**b**) HA solution (c_f_ = 1000 mg/L), and (**c**) oil emulsion (c_f_ = 100 mg/L).

**Figure 7:**
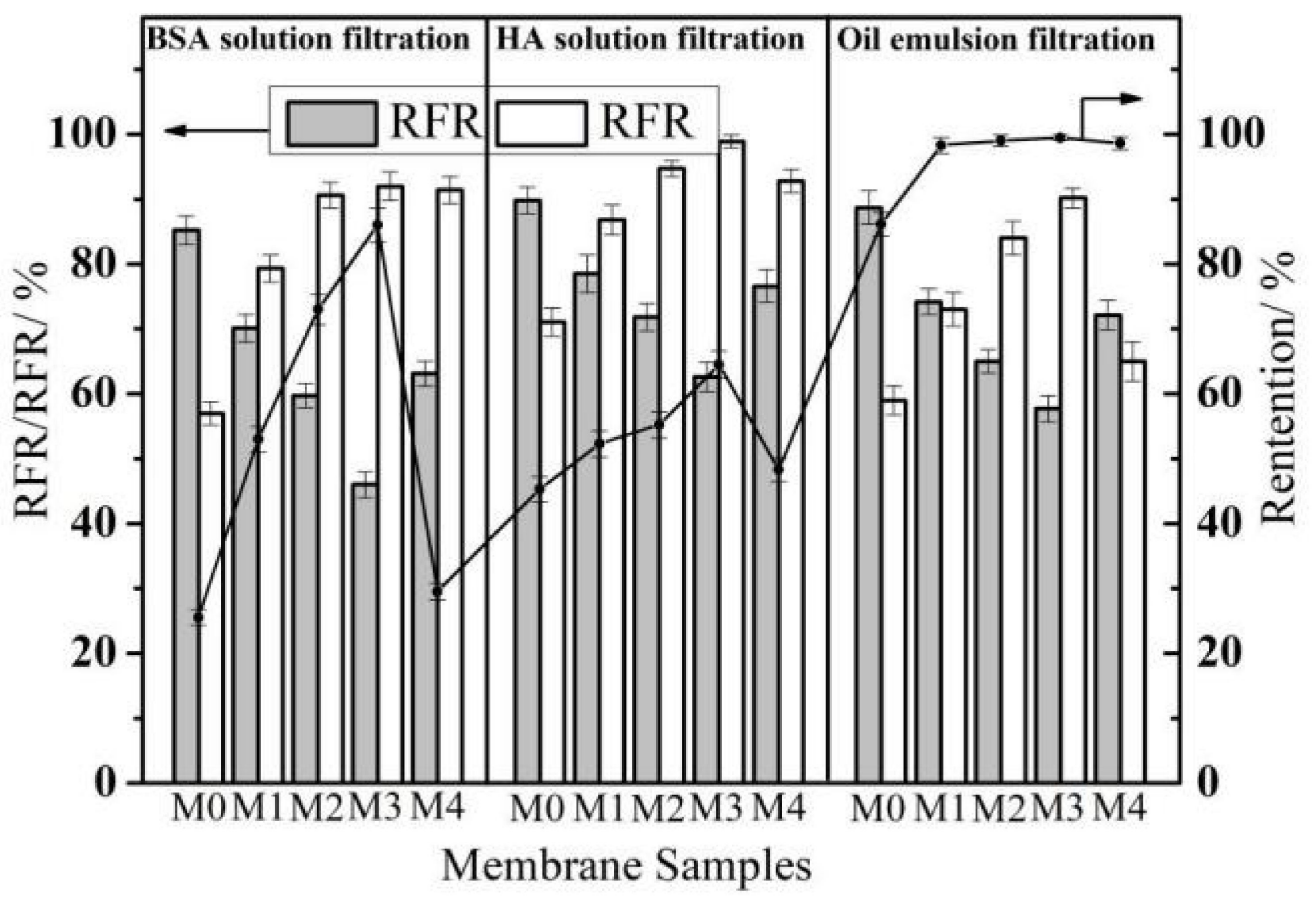
The relative flux decay (RFD), the relative flux recovery (RFR), and the retention rate (R) from the filtration of BSA solution, HA solution, and oil emulsion.

To further confirm that the modified PVDF membranes became more resistant to organic pollutants during membrane separation, all the prepared membranes were examined for their performance in the filtration of BSA solution, HA solution, and oil (n-hexadecane) emulsion. The typical results in the evolution of the permeate flux with filtration time are presented in Figure 6a–c and other process-performance information are shown in Figure 7.

It can be found in Figure 6 that, in the first 30 min of operation for pure water filtration, the water fluxes (denoted as *J*_0_) for each type of prepared membrane were relatively stable and constant. During the following 2 h filtration (time 30 to 150 min) of the BSA solution, HA solution, or oil emulsion, the permeate fluxes for each type of the prepared membranes all declined, more rapidly and significantly in the initial stage, and then gradually stabilized with the filtration time. However, the experimental results clearly show that the largest decline occurred for the PVDF membrane (M0). At the end of the 2 h filtration operation, it reached a relative flux decay (RFD) at 85.2% for the BSA solution, 89.8% for the HA solution and 88.7% for the oil emulsion. In contrast, all the modified PVDF membranes exhibited much lower flux decline rates in the filtration process. The RFD for M1, M2, M3, and M4 was at 70.1%, 59.7%, 46.0%, and 63.1% in the BSA solution filtration, at 78.5%, 71.8%, 62.6%, and 76.6% in the HA solution filtration, and at 74.2%, 65.0%, 57.7%, and 72.1% in the oil emulsion filtration, respectively. Particularly, the M2 and M3 membranes had the highest J_0_ but lowest RFD (see Table 2). It is also interesting to note that the retention rate of the M2 and M3 membranes was also much higher than the unmodified M0 membrane (see the last column in Table 2) for all the three types of organic compounds in the feeds. Both the higher water flux and the greater retention rate undoubtedly support the conclusion that the modified membranes had greatly improved anti-fouling performance compared to the unmodified membrane. The M4 membrane however did not provide a high retention rate in the experiments. One of the identified reasons is that the copolymer with a very high amount of hydrophilic and oleophobic contents did not seem to blend well with PVDF to obtain a homogeneous casting solution and hence more uniform membrane pore size, as also mentioned in early sections. Although more masses of BSA, HA, or oil were rejected by M2 and M3 than M0, the rejected compounds did not seem to attach to these modified membranes to significantly reduce their water flux. Hence, the modified membranes may have developed some mechanisms (due to their hydrophilic and oleophobic feature), such as a physical or a repulsive energetic barrier, that prevented the rejected compounds from adsorbing directly onto the membrane surfaces [54,55]. This speculation was also supported by the observed performance in the water flux recovery after the BSA solution, HA solution, or oil emulsion filtration and the membrane was cleaned. As shown in Figure 6 (during time 150 to 180 min) and given in Figure 7 for the recovered membranes in their pure water fluxes (*J*_1_), the relative flux recovery (RFR) of membrane M0 was only at 57.0% after cleaning in BSA solution filtration, which is in sharp contrast to the RFR at 79.3%, 90.6%, 92.0%, 91.4% for the modified membranes of M1, M2, M3, and M4, respectively. Similarly, the RFR of M0 was at 71.1% after cleaning in HA solution filtration and 59.0% in oil emulsion filtration. In comparison, the RFR of the membranes of M1, M2, M3, and M4 was much higher, at 86.8%, 94.7%, 98.9%, and 92.8% in HA solution filtration, and at 73.0%, 84.0%, 90.2%, 65.0% in oil emulsion filtration, respectively. In other words, the retained compounds by the modified PVDF membrane surfaces were more easily removed by water washing than those on the unmodified PVDF membranes. Particularly, membrane M3 that showed the highest hydrophilicity and oleophobicity also achieved the highest water flux, lowest water flux decay, and highest water flux recovery. Also, the cummulative permeate volume for the various prepared membranes during the firtration of BSA solution, HA solution or oil emulsion (time 30–180 min) can been seen in Figure S1. Due to the membrane fouling, the steep intial decline of the *J*/*J*_0_ value was followed by a gradual decline. While M3 also showed lowest reduction and highest cummulative permeate volume. After cleaning, the *J*/*J*_0_ value of the modified membranes recovery to a higher level as compare to the substrate membrane M0. Thus, the presence of the additive polymer-P(St-*co*-MAA)-*g*-fPEG in the modified PVDF membrane can indeed effectively enhance the anti-fouling performance of the prepared membranes for the representative organic pollutants of BSA, HA, and oil (n-hexadecane) in aqueous solutions.

In the literature, Rajasekhar et al. reported the study in a modified PVDF membrane by blending with synthesized functional additive and the highest flux recovery (RFR) achieved was about 78% for BSA solution filtration and 80% for oil–water emulsion filtration [56]. In more recent research, Li et al. reported the modification of a PVDF membrane by blending nanocomposite BiOCl for BSA solution filtration. They reported the highest membrane flux recovery rates (RFR) at 85% for BSA filtration [57]. In our study, the modified membrane M3 showed very promising anti-fouling performance, with RFR value 92%, 98.9%, and 90.2% for BSA solution, HA solution, and oil emulsion, respectively. The results appear to be better than or comparable to those best results reported in the literature.

**Figure 8:**
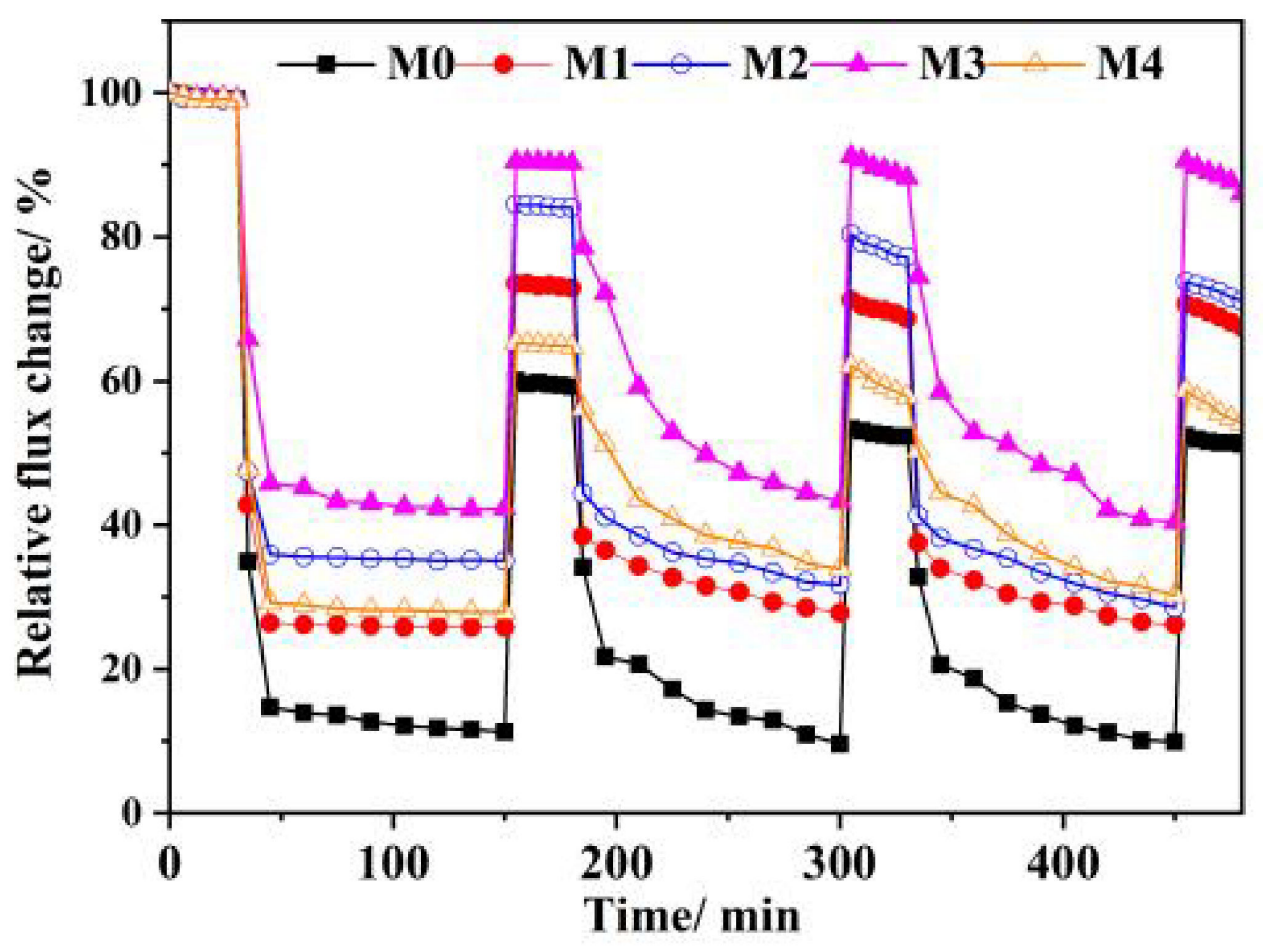
Relative water flux changes with filtration time for the various prepared membranes during the filtration of oil emulsion (c_f_ = 100 mg/L) for three consecutive cycles.

Since oil, which usually has extremely low surface tension (at the range of 18–30 mN/m, significantly lower than most membrane materials), can often be considered as one of the most difficult organic foulants which foul almost all membranes [58], the prepared membranes were further tested in the oil emulsion filtration under similar conditions as before for three consecutive cycles. The obtained results are shown in Figure 8. 

It is clear from the figure that the unmodified PVDF membrane, M0, had a declined trend of both permeate flux as well as flux recovery rate after each cycle of filtration and cleaning, and the modified membranes, particularly M3, displayed very consistent and much-higher permeate flux and flux recovery rate after the three cycles of filtration and cleaning operation. The results suggest that the method presented in this work can be used to develop novel membranes that may have great potential for practical applications.

## 4. Conclusions

A triple-component copolymer additive of P(St_x_-*co*-MAA_y_)-*g*-fPEG_z_ with two compositions of St:MAA(=fPEG) in 4:1 or 1:1 was successfully synthesized by a simpler method and with cheaper materials. The additive polymer provided components for good compatibility with PVDF base polymer as well as hydrophilic and oleophobic surface properties desired for the modified PVDF membranes, and had great effect on the prepared membranes’ properties and anti-fouling performance. The results showed that the prepared PVDF membrane (M3) provided the most desired properties and performance under the conditions examined. Greatly improved surface hydrophilicity (water contact angle reduced from about 80° for the unmodified PVDF membrane M0 to as low as 48.80° for the modified PVDF membrane M3) was achieved. The underwater oil contact angles for the modified membranes were also significantly enhanced, with M3 reaching as high as 160°, essentially into the super-oleophobic range. Experimental results indicated that the developed membrane can reduce BSA adsorption on the membrane by up to 93% in comparison with that of the unmodified PVDF membrane. The filtration experiments for BSA solution, HA solution, and oil/water emulsion confirmed that the obtained novel membrane can perform very well, with much higher water flux and separation rate, but lower flux decay and greater flux recovery after cleaning. Furthermore, the developed membrane with enhanced anti-fouling performance will have great potential for practical applications in water and wastewater treatment, particularly in the oil–water separation field.

## Figures and Tables

**Table 1:** The compositions and some properties of the prepared membranes.

Parameter	Types of Membranes Samples				
	M0	M1	M2	M3	M4
Composition of additive polymer	-	P(St_x_-*co*-MAA_y_)-*g*-fPEG_z_ (x:y = z at 4:1)	P(St_x_-*co*-MAA_y_)-*g*-fPEG_z_ (x:y = z at 4:1)	P(St_x_-*co*-MAA_y_)-*g*-fPEG_z_ (x:y = z at 1:1)	P(St_x_-*co*-MAA_y_)-*g*-fPEG_z_ (x:y = z at 1:1)
PVDF:Additive ratio (in weight)	10:0	9:1	7:3	9:1	7:3
Total content of PVDF and additive in casting solution (wt%)	18	18	18	18	18
PVP in casting solution (wt%)	5	5	5	5	5
NMP in casting solution (wt%)	77	77	77	77	77
Non-solvent coagulant	H_2_O	H_2_O	H_2_O	H_2_O	H_2_O
Measured porosity (%)	60.8	64.2	72.0	78.9	73.5
Measured pore size (μm)	0.229	0.279	0.320	0.424	0.302
Measured initial water contact angle (°)	79.3 ± 2.3	63.7 ± 1.6	61.7 ± 1.2	48.8 ± 1.0	59.4 ± 1.2
Measured oil contact angle under water (°)	135.1 ± 1.8	144.9 ± 2.0	154.0 ± 2.3	160.7 ± 3.2	154.2 ± 2.2
Tensile stress (MPa)	1.58 ± 0.12	1.52 ± 0.11	0.98 ± 0.05	1.08 ± 0.1	0.67 ± 0.08
Breaking elongation (%)	57.10 ± 2.9	56.03 ± 2.1	19.11 ± 0.80	33.94 ± 1.5	19.46 ± 0.75

**Table 2:** XPS analysis results on C1s core-level spectrum of each membrane resolved into different binding compositions.

Types of Membranes	Membrane Surface Compositions (Area %)				
	C–C (284.8 eV)	C–H (286.1 eV)	C–O (288.1 eV)	C–F_2_ (290.7 eV)	C–F_3_ (291.6 eV)
M0	14.00%	46.38%	3.42%	35.95%	0
M1	31.04%	39.98%	6.88%	21.97%	1.20%
M2	36.02%	38.70%	6.69%	12.89%	4.17%
M3	35.12%	37.73%	5.22%	20.58%	1.30%
M4	40.60%	34.60%	5.36%	13.06%	6.36%

## Data Availability

Not applicable.

## Supplementary Materials

The following are available online at https://www.mdpi.com/article/10.3390/membranes11120951/s1, Figure S1: Normalized flux with cumulative permeate volume for the various prepared membranes during the filtration of (a) (c_f_ = 1000 mg/L), (b) HA solution (c_f_ = 1000 mg/L) and (c) oil emulsion (c_f_ = 100 mg/L).
