# Supplementary material for: Highly Effective Anti-Organic Fouling Performance of a Modified PVDF Membrane Using a Triple-Component Copolymer of P(Stx-co-MAAy)-g-fPEGz as the Additive
Source: Membranes (Basel). 2021 Nov 30;11(12):951. doi: 10.3390/membranes11120951 (PMC8707838; doi:10.3390/membranes11120951)
Supplement: Supplementary file 1 [file membranes-11-00951-s001.zip › membranes-1434797-supplementary.pdf]

Highly effective anti-organic fouling performance of a modified PVDF membrane using a triple-*co*mponent copolymer of P(St$_x$-*co*-MAA$_y$)-*g*-fPEG$_z$ as the additive

X.J. Zhou[1,2,3], Y.Z. Sun[1,2], S.S. Shen[1,2,3], Y. Li[1], R.B. Bai[1,2,3] *

1 Center for Separation and Purification materials &Technologyies, Suzhou University of Science and Technology, Suzhou 215009, China, zhou-xiaoji@163.com (X.Z.); sunyizhuo2019@163.com (Y.S.); shusushen@mail.usts.edu.cn (S.S.); liyan370403@163.com (Y.L.);

2 School of Environmental Science and Engineering, Suzhou University of Science and Technology, Suzhou 215009, China

3 Jiangsu Collaborative Innovation Center for Technology and Material of Water Treatment, Suzhou 215009, China

*Correspondence: ceebairb@live.com

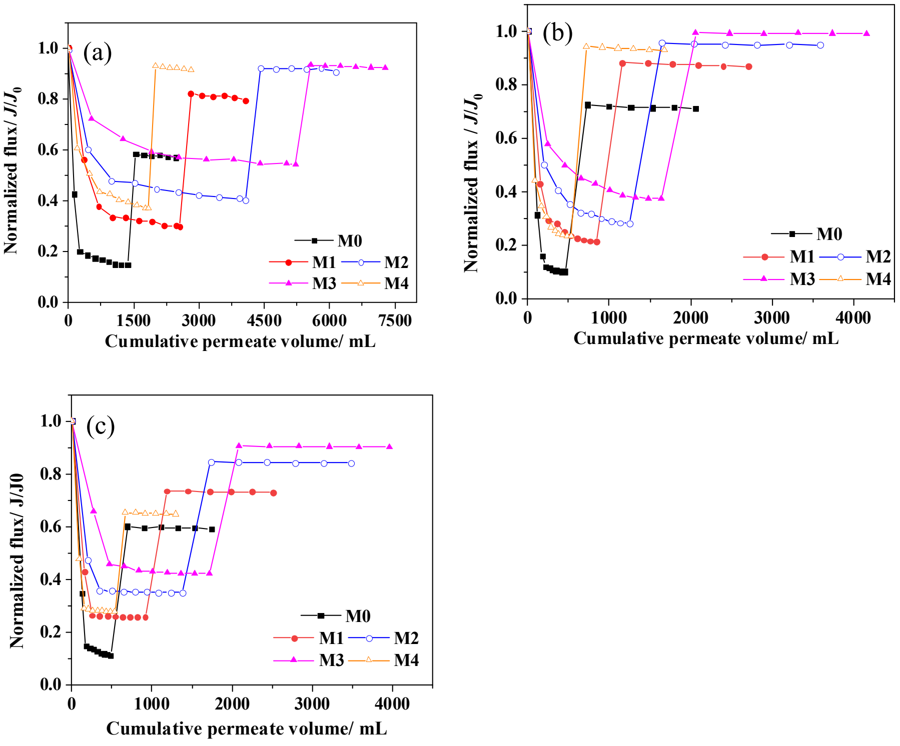

Figure S1 Normalized flux with cumulative permeate volume for the various prepared membranes during the filtration of (a) (c_f = 1000 mg/L), (b) HA solution (c_f = 1000 mg/L) and (c) oil emulsion (c_f = 100 mg/L).
